# Evaluation of isolated left ventricular noncompaction using cardiac magnetic resonance tissue tracking in global, regional and layer-specific strains

**DOI:** 10.1038/s41598-021-86695-0

**Published:** 2021-03-30

**Authors:** Jiamin Zhang, Mengchun Jiang, Chao Zheng, Hui Liu, Yangyu Guo, Xingzhi Xie, ZhiMin Zou, Xiaoyue Zhou, Liming Xia, Meichen Luo, Mu Zeng

**Affiliations:** 1 Department of Radiology, The Second Xiangya Hospital, Central South University, Changsha, Hunan China; 2 Department of Radiology, The First People’s Hospital of Changde City, Changde, Hunan China; 3 MR Collaboration, Siemens Healthineers Ltd., Shanghai, China; 4 Circle Cardiovascular Imaging Inc., 800 5 Ave SW#1100, Calgary, AB T2P 3T Canada

**Keywords:** Cardiovascular diseases, Cardiology

## Abstract

We used cardiac magnetic resonance tissue tracking (CMR-TT) to quantitatively analyze the global, regional and layer-specific strain of isolated left ventricular noncompaction (ILVNC). Combined with late gadolinium enhancement (LGE), we initially explored the effect of focal myocardial fibrosis on myocardial strain. CMR was performed in 63 patients with ILVNC and 52 patients without ILVNC (i.e., the control group). The ILVNC group was divided into an LGE(+) group (29 patients) and an LGE(−) group (34 patients) according to the presence or absence of late gadalinum enhancement (LGE). CVI42 software was used to measure global and regional (basal, middle, apical) radial strain (RS), circumferential strain (CS), longitudinal strain (LS), subendocardial LS and subepicardial LS. The basal–apical strain gradient was defined as the apical mean strain minus the basal mean strain. We then compared differences between these strain parameters. The subendocardial-subepicardial LS gradient was defined as the maximum subendocardial LS minus the subepicardial LS. Compared with the control group, the global and regional RS, CS, LS and the subendocardial, subepicardial LS of the ILVNC group were significantly diminished (*P* < 0.01). Compared with the LGE(−) group, the global and regional RS, CS, LS and the subendocardial, subepicardial LS of the LGE(+) group were significantly diminished (*P* < 0.05). In the ILVNC group, the basal–apical CS and LS gradient, and the subendocardial-subepicardial LS gradient were significantly lower than those in the control group (*P* < 0.01). There were significant differences in myocardial strain between patients with and without ILVNC. ILVNC revealed a specific pattern in terms of strain change. The myocardial strain of the cardiac apex and endocardium was significantly lower than that of the cardiac base and epicardium, respectively. Myocardial strain reduction was more significant in ILVNC patients with focal myocardial fibrosis.

## Introduction

Isolated left ventricular noncompaction (ILVNC) is a congenital left ventricular myocardial structural abnormality with a complex pathogenesis and pathophysiological background. The clinical manifestations are nonspecific and the prognosis varies^1,2^. We have observed that some patients with ILVNC will show LGE(+). It often represents myocardial fibrosis, so we explored whether myocardial fibrosis affects strain. Cardiovascular magnetic resonance (CMR) imaging with late gadolinium enhancement (LGE) has been used to detect myocardial fibrosis, and it is currently the most commonly used method for detecting focal myocardial fibrosis. LGE is used in diagnosis, differential diagnosis, and judgment of disease prognosis and risk stratification. LGE is frequently present in ILVNC patients, and previous studies have associated LGE with systolic dysfunction and major adverse cardiovascular events^3–5^. The accurate assessment of cardiac function can help to better assess pathogenic conditions and guide effective treatment.

The evaluation of cardiac function usually uses the left ventricular ejection fraction (LVEF). However, LVEF can only evaluate global myocardial function. Myocardial strain can be used to evaluate changes in local myocardial motion. Previous studies have shown that patients with cardiomyopathy, including ILVNC, have reduced systolic function and strain when LVEF is normal^6^. Therefore, myocardial strain can more accurately evaluate changes in cardiac function than LVEF. Correlation between CMR tissue tracking (CMR-TT) and speckle tracking echocardiograph (STE) was modest and agreement was not optimal due to systematic bias regarding GLS and GCS. Consequently, CMR-TT and STE should not be used interchangeably for myocardial strain evaluation^7^. This article focuses on CMR strain analysis and no longer compares them.

Cardiac magnetic resonance tissue tracking (CMR-TT), which is based on the conventional cine sequence, has become a commonly used technique to assess myocardial strain. Clinically, accurate assessment of heart function helps to better assess the severity of the disease and guide effective treatment. Strain evaluates cardiac function more accurately than EF. The myocardial strain characteristics of patients with ILVNC is still unclear. Therefore, the main purpose of this article is to analyze the global, regional and layer-specific strains of the ILVNC group and the control group to obtain the myocardial strain pattern of ILVNC patients, so as to assess changes in cardiac function more accurately. Myocardial fibrosis often affects cardiac function, so we hypothesis that the myocardial strain of ILVNC patients with LGE(+) decreased more significantly than that of LGE(−), which will be verified in the text. The relationship between LGE(+) and myocardial strain in ILVNC patients will be analyzed.

## Materials and methods

### Study population

A retrospective study was conducted of 63 patients with ILVNC diagnosed in the Second Xiangya Hospital of Central South University from August 2016 to January 2020. The inclusion criteria were as follows^8–10^: (1) the left ventricular wall showed a bilayer appearance of compacted myocardium (C) and noncompacted myocardium (NC), (2) a noncompacted myocardium was evident with an obvious trabecular meshwork and deep intertrabecular recesses, (3) the end-diastolic ratio of noncompacted to compacted myocardium (NC/C ratio) was > 2.3 involving at least three segments^8^, (4) no other cardiac abnormalities were present. Once successfully enrolled, patients with ILVNC were categorized into LGE(+) and LGE(−) groups. Fifty-two age- and sex-matched healthy individuals were enrolled in the control group. The inclusion criteria for controls were as follows: (1) no history of cardiovascular disease, (2) chest radiograph, electrocardiogram and echocardiography findings were normal, (3) CMR showed normal cardiac function.

This study was approved by ethics committee of the Second Xiangya Hospital of Central South University, and informed consent was signed by all participants. All methods were carried out in accordance with relevant guidelines and regulations.

### Magnetic resonance imaging protocols

Scanning were performed on a 3 T scanner (MAGNETOM Skyra, Siemens Healthineers) with 18-channel body coil scan protocols. The scanning sequence was carried out as follows:

1. A cine balanced steady-state free processing (bSSFP) sequence with segmented acquisitions, which collected left ventricular two-chamber, four-chamber, and short-axis images at the end of inspiration, with 25 retrospectively calculated phases per cardiac cycle. The scanning parameters were as follows: repetition time, 3.2 ms; echo time, 1.43 ms; flip angle, 44°; temporal resolution, 40 ms; field of view, 320 mm × 400 mm; acquisition matrix, 126 × 224; slice thickness, 8 mm with no slice gap.
2. LGE with gadopentetate dimeglumine as the contrast agent at a dose of 0.2 mmol per kilogram of body weight. After the contrast agent had been injected, 20 ml of normal saline was injected. All patients in the ILVNC group and control group were injected with the contrast agent. Images were taken 10 minutes after the injection of contrast agent. Scan parameters were as follows: repetition time: 2.8 ms; echo time: 1.3 ms; flip angle: 40°; field of view: 320 mm × 400 mm; acquisition matrix: 125 × 256.

### Image analysis

#### Conventional cardiac function assessment

All conventional cardiac function parameters were obtained using CVI42 post-processing software (Circle Cardiovascular Imaging Inc, version 5.9.3). The parameters included LVEF, left ventricular end-diastolic volume index (LVEDVI), left ventricular end-systolic volume index (LVESVI), left ventricular stroke volume index (LVSVI), and left ventricular myocardial mass index (LVMMI). Index mean parameters were indexed to body surface areas.

#### Trabeculation and late gadolinium enhancement

According to the 17-segment method for left ventricular myocardium established by the American Heart Association, we measured the thickness of the compacted (C) layer, the thickness of the noncompacted (NC) layer, and the NC/C ratio on the end-diastolic short-axis cine sequence image of 1–16 segments. Because the measurement error in section 17 is relatively large, quantitative measurements do not include this section. Segments with an NC/C ratio greater than 2.3 were defined as noncompacted segments. We calculated mean values for C, NC, and NC/C in the noncompacted segments. The LGE images were visually inspected for the presence or absence of LGE by two experienced radiologists. If the results were inconsistent, a third radiologist was introduced and asked to judge the results in a blinded manner. Each radiologist studied CMR for more than 3 years.

#### Strain analysis

**Figure 1:**
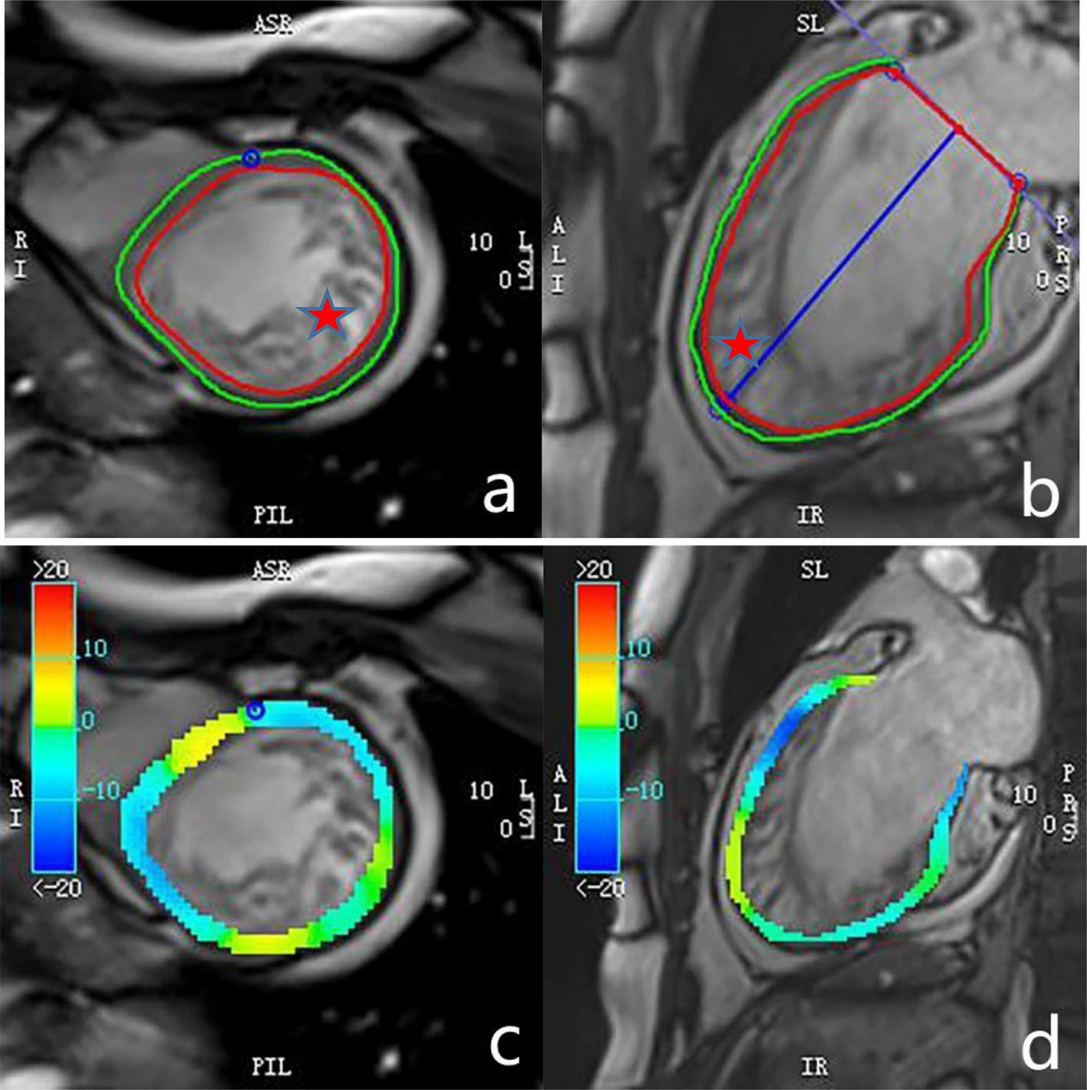
A patient with isolated left ventricular noncompaction and the post-processing of left ventricle strain. A large number of loose trabeculae and deep recesses were evident in the left ventricular lateral wall and apical segment of this patient (mark with an asterisk). We delineated the endocardium and epicardium in the short-axis and long-axis cine images, and we marked the left and right chamber boundary points in the short-axis image (**a**,**b**). Circumferential (**c**) and longitudinal (**d**) strain (%) pseudo-color maps.

**Figure 2:**
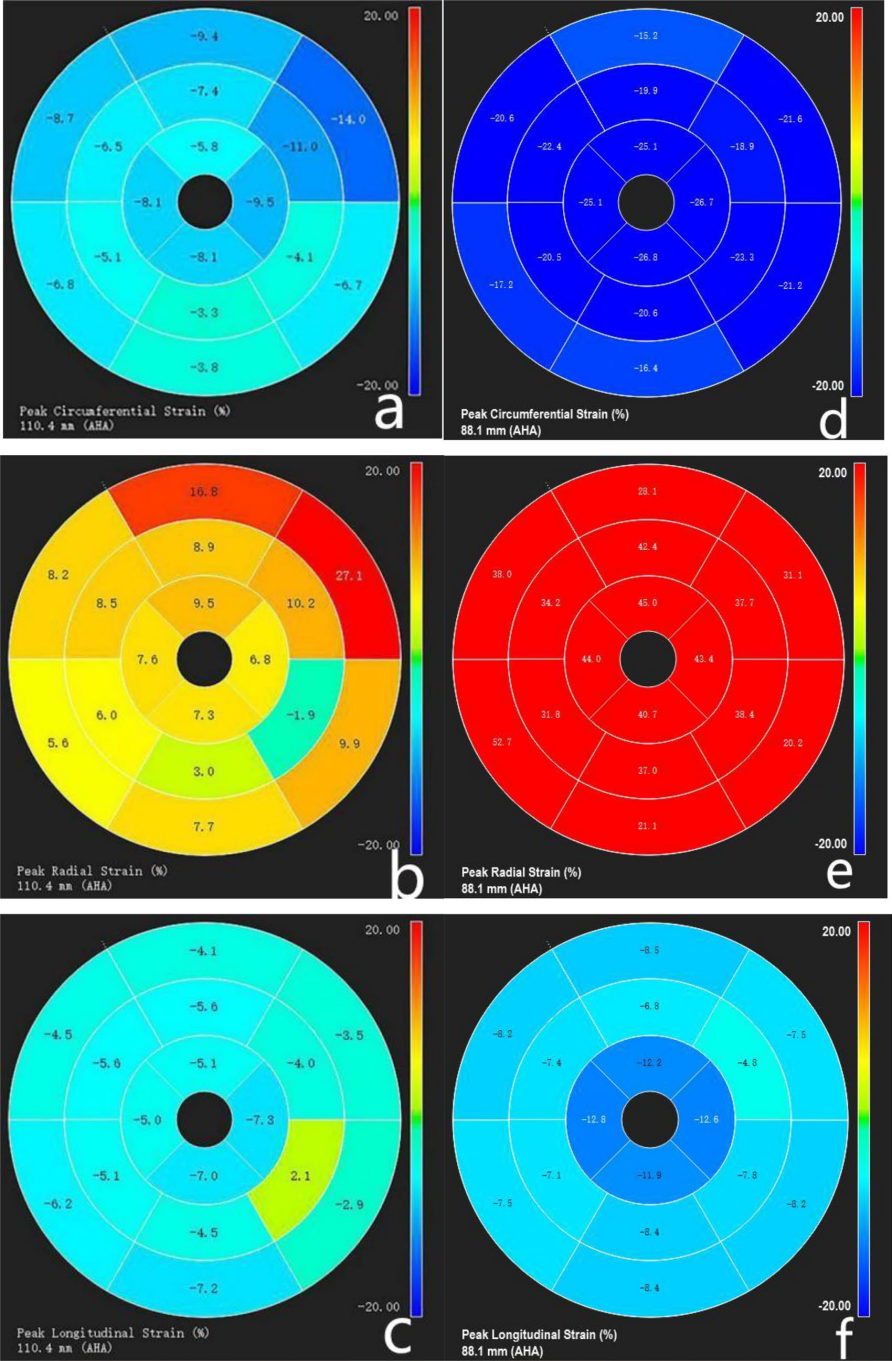
The bull’s eye diagram of myocardial strain in a patient with isolated left ventricular noncompaction (ILVNC) that show diagrams of global circumferential strain (**a**), radial strain (**b**), and longitudinal strain (**c**). The other three picture are GCS, GRS and GLS of a control respectively (**d**–**f**). Compare to controls, the global and segmental strains in ILVNC patients were reduced, and the directions were different.

All strain parameters were obtained using CVI42 post-processing software. In the tissue tracking module, we introduced the left ventricle short-axis, two-chamber, and four-chamber long-axis cine images. The subendocardium and subepicardium were delineated at the end of diastole and end-systolic period, and the left and right ventricle demarcation points were marked manually (Fig. 1). Myocardial strain parameters were obtained using the software’s automatic post-processing setting, including left ventricular myocardium global, regional and segmental radial strain (RS), circumferential strain (CS), longitudinal strain (LS), layer-specific LS, the strain curve, and a 16-segment bull's eye diagram of each parameter (Fig. 2). The apex (segment 17) was excluded from strain analysis.

### Statistical analysis

Statistical analysis was performed using IBM SPSS Statistics (IBM Corporation, version 22.0). Data were tested for normality using the Kolmogorov–Smirnov method. Continuous data are expressed as the means ± standard deviations (SDs). Comparisons of two normally distributed variables were performed using an independent sample student *t*-test. The Mann–Whitney U test was used to compare two variables that did not conform to a normal distribution. The Spearman method was used to test the correlation between strain parameters and LVEF or LVEDVI. Logistic regression models and receiver operating characteristic (ROC) curves were used to evaluate the efficacy of myocardial strain parameters in predicting LGE. Bland–Altman plots were used to evaluate intra- and interobserver reproducibility. A *P*-value less than 0.05 was considered to be statistically significant.

## Results

### Demographics and conventional cardiac function

The ILVNC group (63 patients) comprised 44 men (69.8%) and 19 women (30.2%), and the mean age was 44.44 years ± 14.4 SD. The control group (52 patients) comprised 36 men (69.2%) and 16 women (30.8%), with a mean age of 44.10 years ± 15.7 SD.

Compared with the control group, LVEF and LVSVI in the ILVNC group were significantly lower (P < 0.01), whereas LVEDVI, LVESVI, and LVMMI were significantly higher (P < 0.01). Compared with the LGE(−) group, LVEF and LVSVI in the LGE(+) group were significantly lower (P < 0.01), whereas LVEDVI, LVESVI, and LVMMI were higher (P < 0.05). Baseline characteristics and conventional CMR function parameters are shown in Table 1.

**Table 1:** Baseline characteristics and conventional cardiac magnetic resonance function parameters for 115 study patients.

Parameters^a^	Control group (n = 52)	ILVNC group (n = 63)	P value	ILVNC group		*P* value
				LGE(+) (n = 29)	LGE(−) (n = 34)	
Age, year	44.10 (5.7)^b^	44.44 (14.4)	0.902	48.93 (14.8)	40.62 (13.0)	0.021
**Sex**						
Male	36 (69.2%)	44 (69.8)	0.944	23 (79.3%)	21(61.8%)	0.320
Female	19 (30.2%)			6 (20.7%)	13 (39.2%)	
LVEF, %	62.51 (6.1)	23.72 (13.3)	< 0.01	17.68 (10.9)	28.87 (13.1)	< 0.01
LVEDVI, ml/m^2^	62.30 (12.2)	159.16 (78.3)	< 0.01	180.59 (91.2	140.88 (60.9)	0.044
LVESVI, ml/m^2^	23.50 (7.0)	125.45 (71.2)	< 0.01	150.77 (82.0)	103.85 (52.6)	< 0.01
LVSVI, ml/m^2^	38.80 (8.4)	33.71 (18.2)	< 0.01	29.82 (17.5)	37.03 (18.4)	0.080
LVMMI, g/m^2^	44.26 (9.1)	78.03 (33.3)	< 0.01	88.61 (35.2)	69.00 29.0)	0.026

*ILVNC* isolated left ventricular noncompaction, *LGE* late gadolinium enhancement, *LVEF* left ventricular ejection fraction, *LVEDVI* left ventricular end-diastolic volume index, *LVESVI* left ventricular end-systolic volume index, *LVSVI* left ventricular myocardial mass index, *LVMMI* left ventricular myocardial mass index.

^a^Corresponding values outside parentheses reflect the mean value unless referring to sex (no. of n) or *P*-values.

^b^Values in parentheses reflect ± standard deviation unless otherwise noted.

### Late gadolinium enhancement distribution and trabeculation

**Figure 3:**
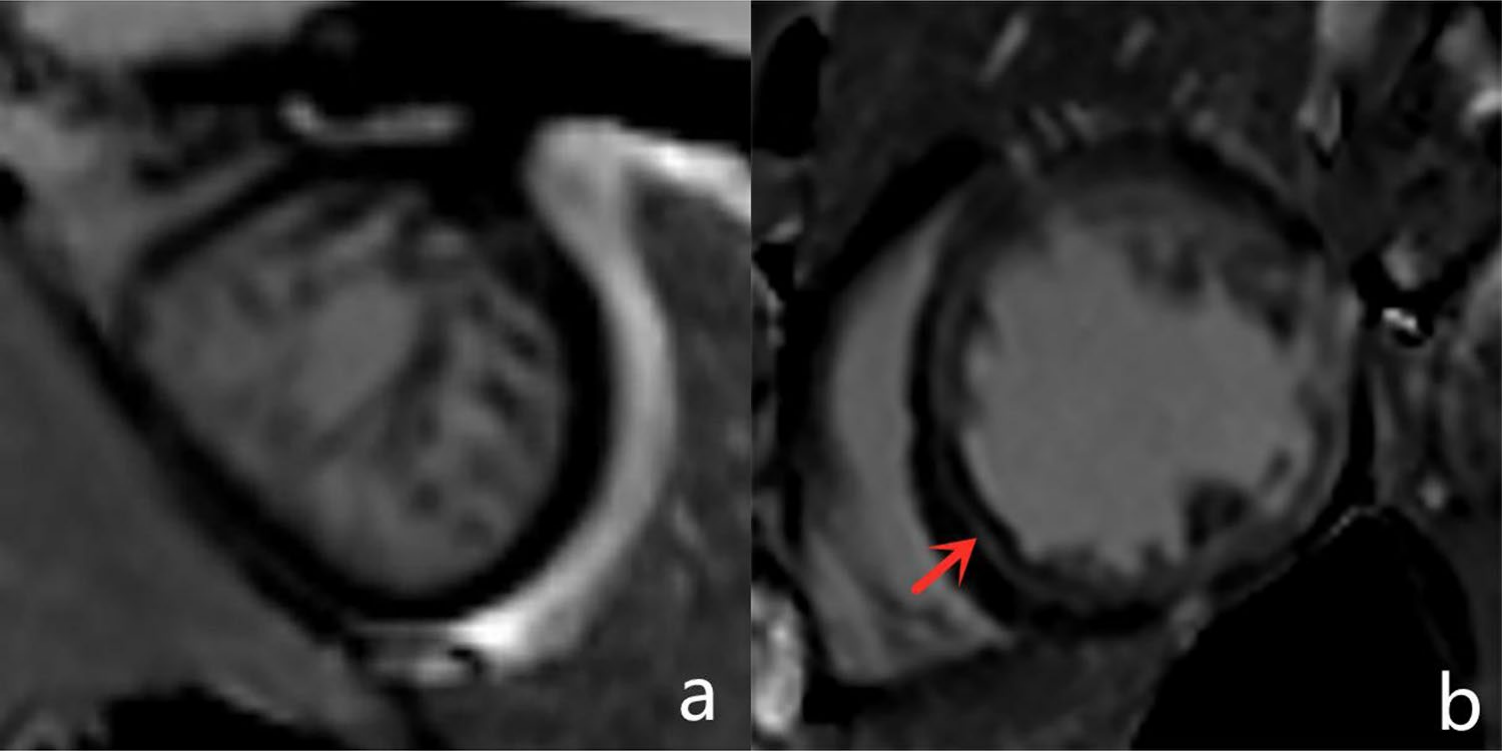
LGE was not seen in the myocardium in a patient with isolated left ventricular noncompaction (ILVNC) (**a**). Linear LGE can be seen in the middle myocardium of the interventricular septum in another patient (**b**). (Indicated by the red arrow).

A total of 29 patients (46.0%) in the ILVNC group presented with LGE. Mid-myocardial LGE was found in 25 patients (86.2%), the most common of which was in the ventricular septum. Subendocardium LGE was found in two patients (6.9%), transmural LGE was found in two patients (6.9%) (Fig. 3).

There were 257 noncompacted segments (NC/C > 2.3) in the ILVNC group, of which the basal, middle, and apical parts accounted for 10.1%, 27.6%, and 62.3%, respectively. There was no significant difference of NC/C between the LGE(+) and LGE(−) groups (3.10 ± 0.5 vs 3.08 ± 0.6; *P* = 0.895).

### Strain parameters and their statistical analysis

Compared to the control group, the global and regional RS, CS, LS and the subendocardial, subepicardial LS of the ILVNC group were significantly diminished (*P* < 0.01). Compared to the LGE(−) group, the LGE(+) group had diminished global and regional RS, CS and LS (*P* < 0.05) as well as diminished subendocardial, subepicardial LS (*P* < 0.01) (Table 2).

**Table 2:** Global, regional, and layer-specific myocardial strain parameters in 115 study patients.

Parameters, %	Control group (n = 52)	ILVNC group (n = 63)	*P* value	ILVNC group		*P* value
				LGE(+) (n = 29)	LGE(−) (n = 34)	
LV global RS	36.68 (7.9)^a^	12.17 (8.6)	< 0.01	8.41 (7.2)	15.4 (8.5)	< 0.01
LV basal RS	43.30 (10.3)	16.97 (11.4)	< 0.01	12.26 (9.7)	21.00 (11.3)	< 0.01
LV middle RS	34.27 (8.8)	11.40 (9.4)	< 0.01	7.48 (7.9)	14.74 (9.4)	< 0.01
LV apical RS	38.49 (12.0)	11.98 (8.9)	< 0.01	8.53 (7.7)	14.93 (9.1)	< 0.01
LV global CS	− 21.43 (2.9)	− 8.33 (4.5)	< 0.01	− 6.46 (4.0)	− 9.93 (4.3)	< 0.01
LV basal CS	− 19.06 (2.8)	− 8.78 (4.3)	< 0.01	− 6.79 (3.5)	− 10.50 (4.2)	< 0.01
LV middle CS	− 21.26 (2.9)	− 8.21 (4.8)	< 0.01	− 6.28 (4.3)	− 9.86 (4.6)	< 0.01
LV apical CS	− 24.54 (3.3)	− 9.08 (5.0)	< 0.01	− 7.13 (4.9)	− 10.73 (4.6)	< 0.01
LV global LS	− 15.65 (2.7)	− 6.67 (3.5)	< 0.01	− 5.52 (3.3)	− 7.64 (3.5)	0.017
LV basal LS	− 13.6 (2.5)	− 6.02 (3.7)	< 0.01	− 4.76 (3.5)	− 7.08 (3.5)	0.011
LV middle LS	− 15.18 (3.2)	− 6.56 (3.8)	< 0.01	− 5.44 (3.2)	− 7.50 (4.0)	0.029
LV apical LS	− 17.91 (2.5)	− 7.96 (4.1)	< 0.01	− 6.50 (3.9)	− 9.20 (4.0)	< 0.01
LV subendocardial LS	− 17.72 (3.1)	− 8.21 (4.7)	< 0.01	− 6.03 (4.0)	− 10.07 (4.5)	< 0.01
LV subepicardial LS	− 16.18 (2.9)	− 7.58 (4.5)	< 0.01	− 5.56 (3.7)	− 9.31 (4.5)	< 0.01

*ILVNC* isolated left ventricular noncompaction, *LGE* late gadolinium enhancement, *LV* left ventricular, *RS* radial strain, *CS* circumferential strain, *LS* longitudinal strain.

^a^Values in parentheses reflect ± standard deviation.

LVEF was correlated with global RS, CS, and LS (r = 0.869, − 0.895, − 0.794, respectively; *P* < 0.01). LVEDVI was also correlated with global RS, CS, and LS (r =  − 0.565, 0.551, 0.463, respectively; *P* < 0.01,).

**Figure 4:**
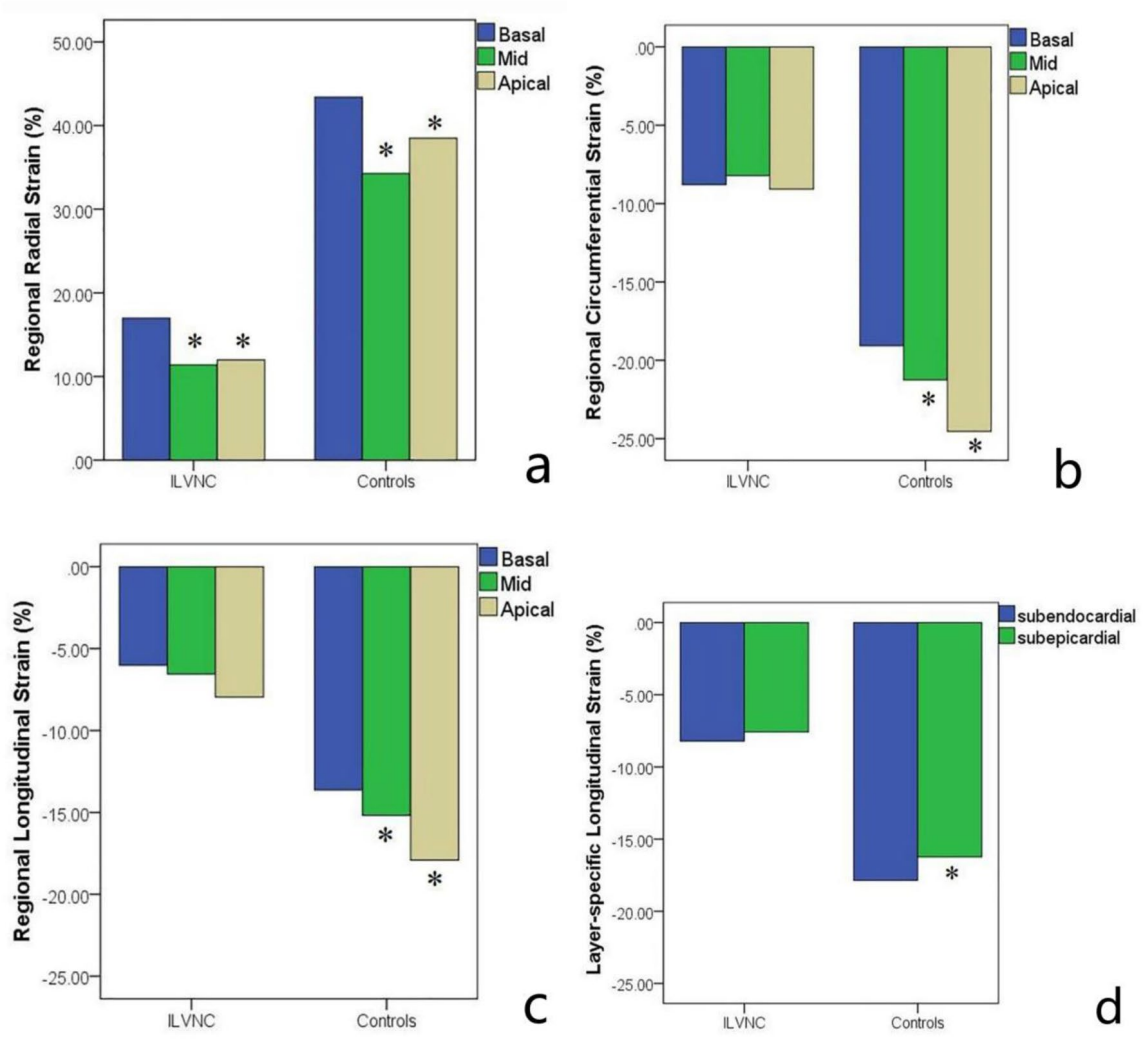
Comparison of basal to apical radial, circumferential, and longitudinal strain values in the isolated left ventricular noncompaction (ILVNC) group and the control group (**a**–**c**), *means compared with the basal strain, *P* < 0.05. Comparison of subepicardial to subendocardial longitudinal strain values in the ILVNC group and the control group (**d**), *means compared with the subendocardial longitudinal strain, *P* < 0.05.

Both CS and LS in the control group increased gradually from the base to the apex (*P* < 0.01). In the ILVNC group, CS and LS did not tend to increase from the base to the apex (*P* > 0.05) (Fig. 4).

The basal–apical strain gradient was defined as the apical mean strain minus the basal mean strain. The basal–apical CS, LS gradient of the ILVNC group was significantly lower than that in the control group (*P* < 0.01) (Table 3).

**Table 3:** Myocardial strain gradient.

Parameters	Control group (n = 52)	ILVNC group (n = 63)	*P* value	ILVNC group		*P* value
				LGE(+) (n = 29)	LGE(−) (n = 34)	
LV basal–apical CS gradient	− 5.47 (2.8)	− 0.30 (2.7)	< 0.01	− 0.34 (2.9)	− 0.26 (2.5)	0.899
LV basal–apical LS gradient	− 4.27 (1.6)	− 1.94 (2.5)	< 0.01	− 1.74 (2.1)	− 2.12 (2.8)	0.542
LV subepicardial-subendocardial LS gradient	− 1.65 (0.9)	− 0.62 (0.8)	< 0.01	− 0.47 (0.7)	− 0.75 (0.8)	0.162

*ILVNC* isolated left ventricular noncompaction, *LGE* late gadolinium enhancement, *LV* left ventricular, *RS* radial strain, *CS* circumferential strain, *LS* longitudinal strain, – not applicable.

^a^Values in parentheses reflect ± standard deviation.

The subendocardial LS was significantly higher than the subepicardial LS in the control group (*P* < 0.05), although there was no difference between the subendocardial and subepicardial LS in the ILVNC group (*P* > 0.05) (Fig. 4). The subendocardial-subepicardial LS gradient was defined as the maximum subendocardial LS minus the subepicardial LS. The subendocardial-subepicardial LS gradient in the ILVNC group was significantly lower than that in the control group (*P* < 0.01) (Table 3).

**Figure 5:**
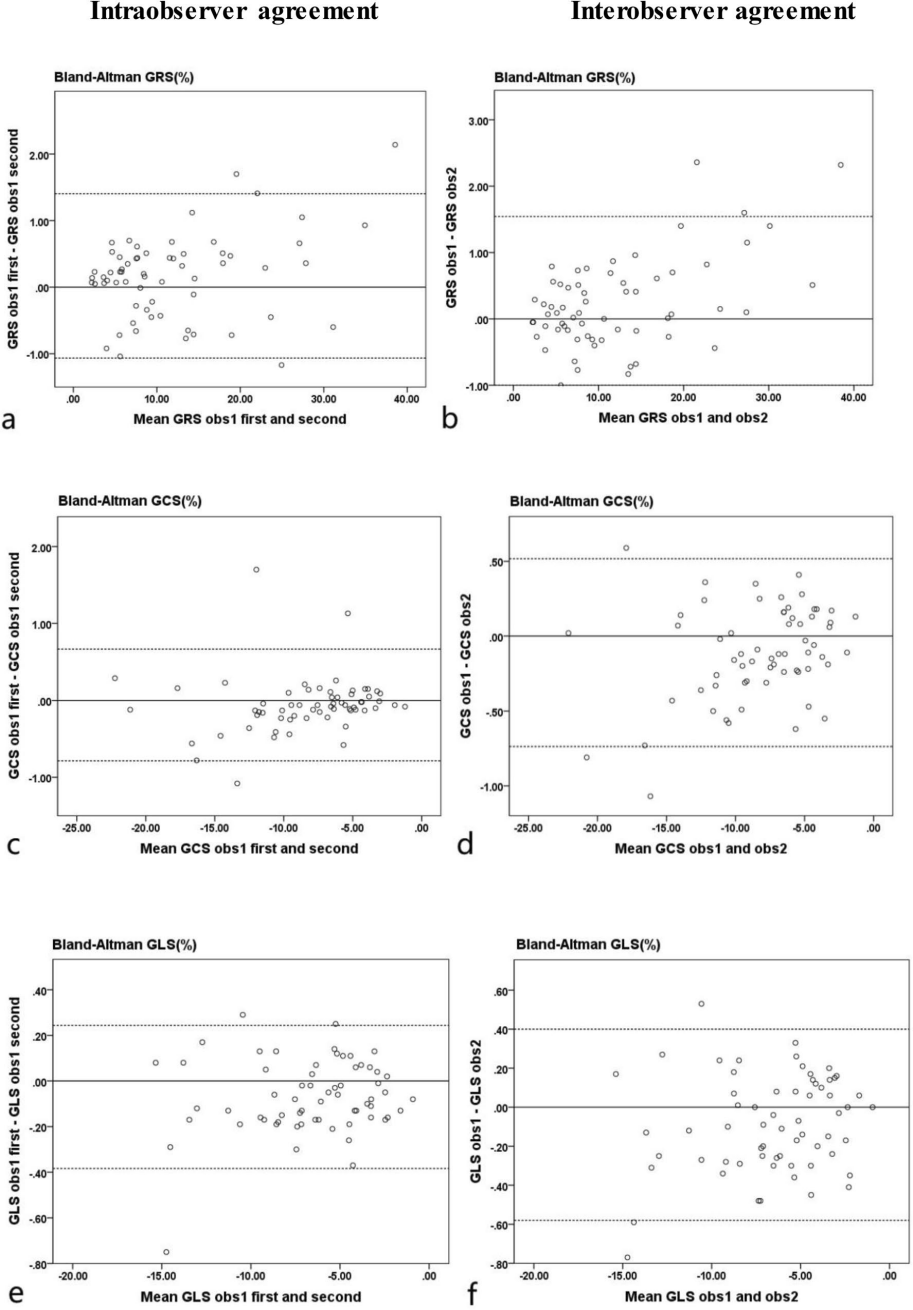
Intra- and interobserver agreements of the patients with isolated left ventricular noncompaction. Bland–Altman plots show the agreement for intraobserver measurements (**a,c,e**), and interobserver measurements (**b,d,f**) for global radial, circumferential, and longitudinal strains. *GRS* global radial strain, *GCS* global circumferential strain, *GLS* global longitudinal strain.

**Figure 6:**
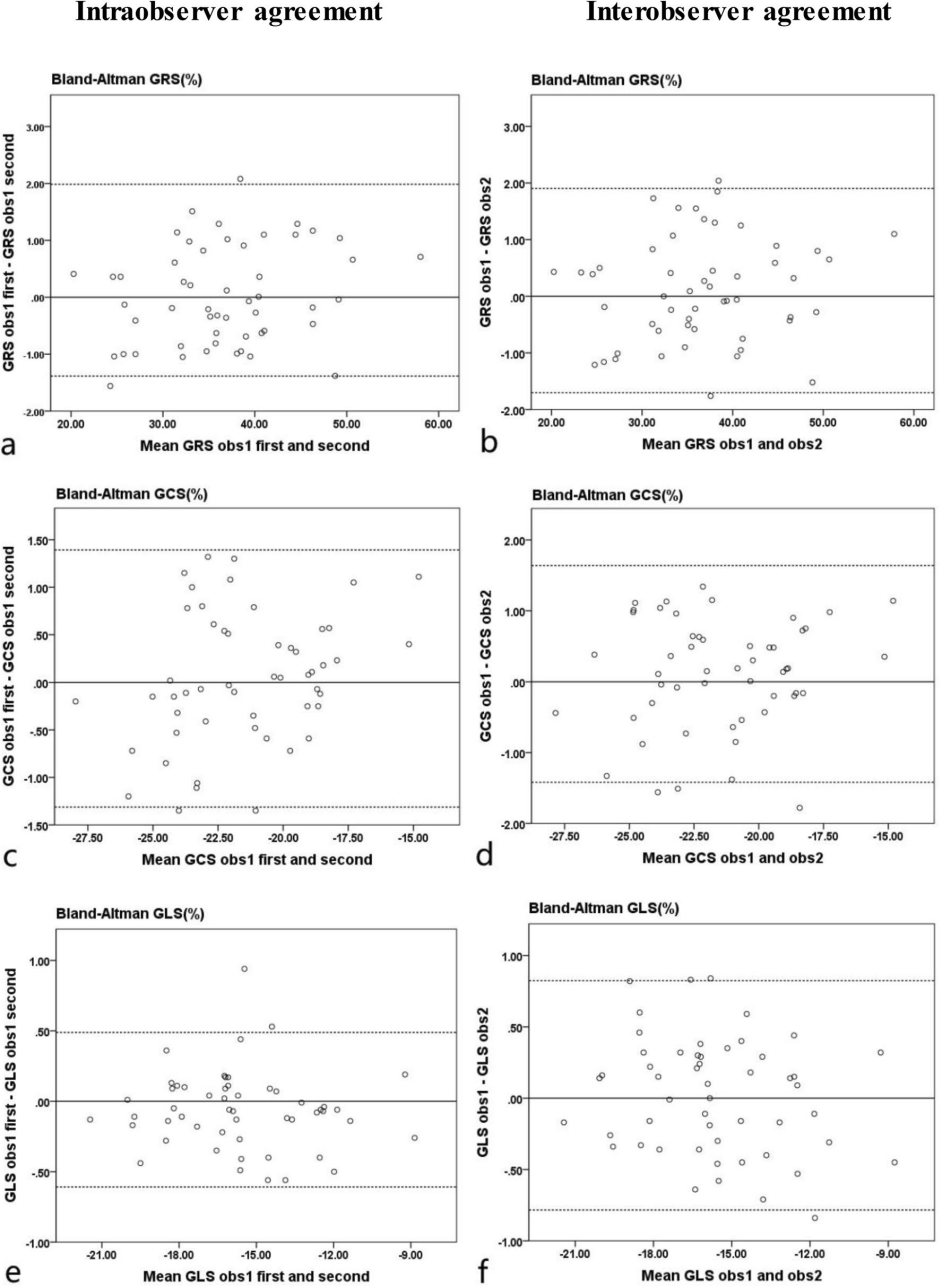
Intra- and interobserver agreements of the control subjects. Bland–Altman plots show the intraobserver measurement (**a,c,e**) and interobserver measurement (**b,d,f**) agreements for global radial, circumferential, and longitudinal strains. *GRS* global radial strain, *GCS* global circumferential strain, *GLS* global longitudinal strain.

The global RS, CS, LS of the ILVNC and control groups were measured within intra- and interobservers. We used the Bland–Altman plots to evaluate the intra- and interobserver reproducibility. The intraobserver and interobserver reproducibility were high for global RS, CS, and LS in the ILVNC (Fig. 5) and control (Fig. 6) groups. There was good to excellent inter- and intraobserver reproducibility for GRS, GLS and GCS in the ILVNC and control groups.

### Value of strain parameters as predictors for LGE

**Figure 7:**
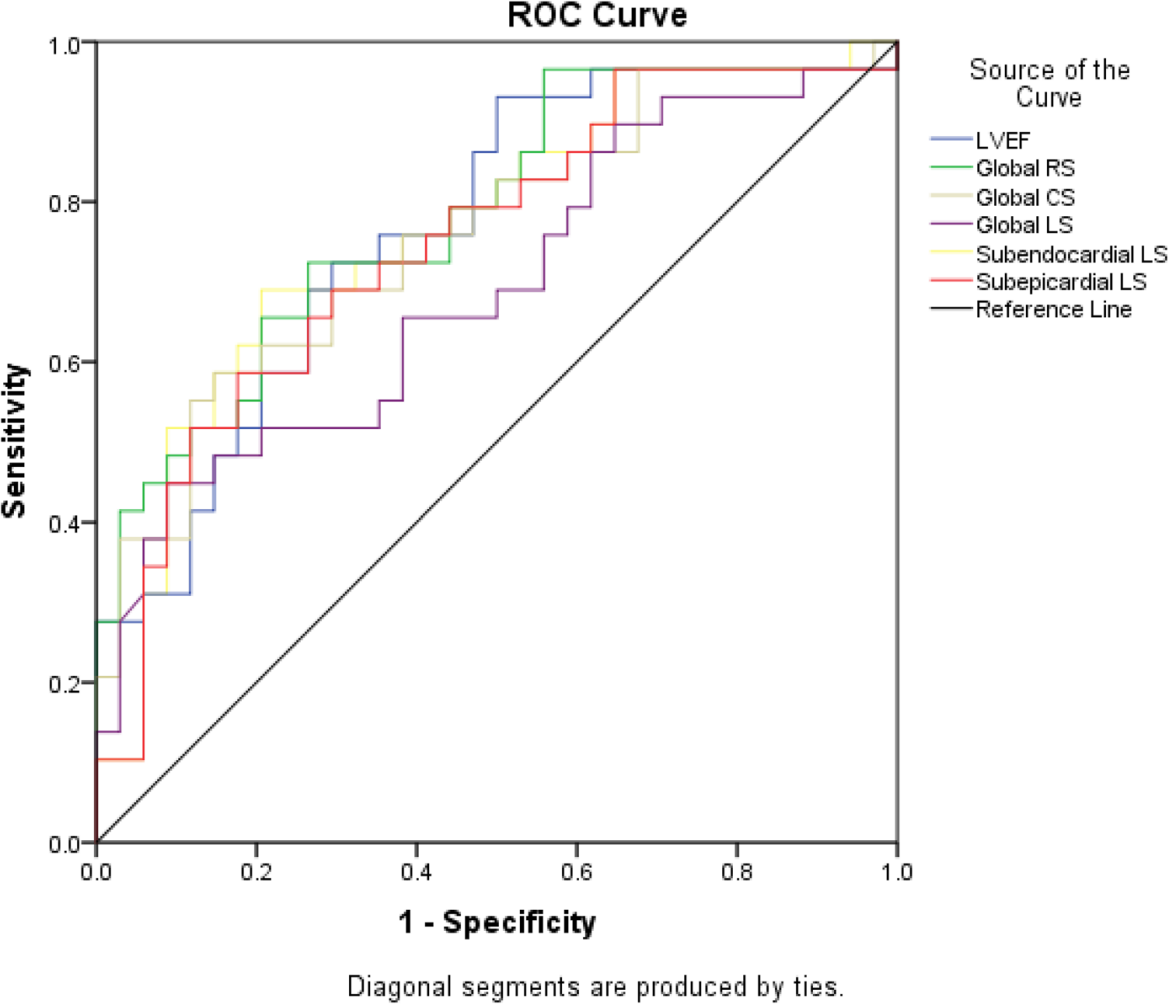
Receiver operating characteristic curves for the univariable analysis of strain parameters can predict LGE. *LVEF* left ventricular ejection fraction, *ROC* receiver operating characteristic, *CS* circumferential strain, *LS* longitudinal strain, *RS* radial strain.

LGE-positive were used as the dependent variable (Y), the Logistic regression model was used to conduct single-factor ROC analysis on LVEF, global RS, CS, LS, subendocardial LS and subepicardial LS. LVEF, (area under the curve [AUC] = 0.767), RS (AUC = 0.802), CS (AUC = 0.759), LS (AUC = 0.690), subendocardial LS (AUC = 0.766) and subepicardial LS (AUC = 0.747) showed predictive value for LGE. (Table 4, Fig. 7).

**Table 4:** Receiver operating characteristic analysis of global and layer-specific strain to detect LGE.

Parameters (%)	AUC	P value	Sensitivity (%)	Specificity (%)	Cutoff value
LVEF	0.767	< 0.01	93.1	50.0	27.5
LV Global RS	0.802	< 0.01	72.4	73.5	8.61
LV Global CS	0.759	< 0.01	58.6	85.3	− 5.96
LV Global LS	0.690	< 0.01	44.8	91.1	− 4.15
LV subendocardial LS	0.766	< 0.01	79.0	79.4	− 6.94
LV subepicardial LS	0.747	< 0.01	58.6	82.3	− 5.55

*AUC* area under the curve, *LGE* late gadolinium enhancement, *LV* left ventricular, *LVEF* left ventricular ejection fraction, *RS* radial strain, *CS* circumferential strain, *LS* longitudinal strain.

## Discussion

The purpose of this study was to explore the strain change patterns of ILVNC by quantitatively evaluating global, regional, and layer-specific myocardial strain. We also evaluated any correlation between LGE and strain in patients with ILVNC.

Studies have suggested that CMR deformation indices including GLS, GCS and strain rate parameters were reduced in patients with LVNC especially in affected midventricular and apical slices and correlated well with parameters of the non-compacted myocardium^11^. In this article, in addition to comparing the global and regional strain of the ILVNC group and the control group, we also performed Layer-specific strain, strain gradient analysis, and what we find is that the subendocardial and subepicardial LS of the ILVNC group were significantly diminished (P < 0.01), the basal–apical CS and LS gradient and the subendocardial-subepicardial LS gradient were significantly lower than those in the control group (P < 0.01). The correlation between myocardial fibrosis and myocardial strain was also analyzed. We find that the global and regional RS, CS, LS, and the subendocardial and subepicardial LS of the LGE(+) group were significantly diminished compared with the LGE(−) group (P < 0.05). We obtained more about the myocardial strain change pattern of ILVNC patients from these analyses.

According to the morphologic and functional characteristics of the heart, ILVNC can be divided into different subtypes, including dilated ILVNC, hypertrophic ILVNC, restrictive ILVNC, and hypertrophic dilated ILVNC^1^. The dilated subtype is the most common, and studies have shown that patients with dilated ILVNC have a worse prognosis than those without dilated ILVNC^12^. In the current study, the LVEDVI of the ILVNC group was significantly higher than that of the control group, and there was a correlation between LVEDVI and the global strain. This finding suggests that the expansion of left ventricular volume is associated with myocardial dysfunction in ILVNC patients.

In the early stages of embryo development, the myocardium gradually undergoes compaction from the subepicardium to the subendocardium, from the base to the apex. Failure of this process leads to abnormally large, disordered trabeculae and deep trabecular crypts, resulting in myocardial noncompaction^13^. Therefore, the apical part is more severely affected than the basal part. In the current study, the apical segments of all patients with ILVNC were involved, and the compacted layer of the myocardium was significantly thinned. Analysis of regional strain found that in the control group, the apical CS and LS were significantly higher than that of the basal part; the reason for this is not clear but might be related to the geometry of the heart^14^.

The longitudinal and circumferential myocardial fibers integrate at the apex. During systole, the radius of curvature of the ventricular wall gradually decreases from the base to the apex, and the stress of the ventricular wall gradually decreases toward the apex. However, in the ILVNC group, there was no statistical difference between the basal and apical CS and LS. To evaluate the trend and extent of strain changes from the base to the apex, we defined the basal–apical strain gradient. The basal–apical CS and LS gradient in the ILVNC group was significantly lower than that in the control group; myocardial function decreased from the base to the apex in a non-homogeneous manner. This finding is consistent with those of Niemann et al.^15^ and Haland et al.^16^, who used tissue Doppler imaging and ultrasound speckle tracking to identify unique strain change patterns in ILVNC. The authors postulated that this was related to the compaction process of the embryonic myocardium from the base to the apex.

CMR-TT can obtain subendocardial and subepicardial strain. The myocardium of the left ventricle is divided into three layers: the subendocardial myocardium, the middle myocardium, and the subepicardial myocardium. The subendocardial and subepicardial myocardium are mainly longitudinally arranged and thereby related to the longitudinal movement of the heart^17,18^. We compared the subendocardial and subepicardial LS in the control group, observing that the subendocardial LS was higher. The presence of this gradient may be related to differences in the focal curvature between the subepicardial and subsubendocardial myocardium^19^. However, in patients with ILVNC, no statistical difference was found between the subendocardial and subepicardial LS. Then we defined the basal–apical strain gradient. The current study results showed that the subendocardial-subepicardial LS gradient of the patients with ILVNC was significantly lower than that of the control group. That is, the degree of subendocardial myocardial involvement was relatively more severe. The subendocardial compacted myocardium was replaced by noncompacted myocardium, and the myocardium was grossly disordered. Therefore, in patients with ILVNC, subendocardial myocardial dysfunction is more pronounced than in the subepicardial myocardium.

In the current study, the global and regional RS, CS, and LS of the LGE(+) group decreased compared to those of the LGE(−) group, demonstrating that myocardial fibrosis can cause limited myocardial movement in different directions. Further layer-specific studies of LS revealed that in patients with ILVNC with LGE (i.e., the LGE(+) group), subendocardial and subepicardial LS was also lower than in patients without LGE (i.e., the LGE(−) group), indicating that the myocardial layer-specific strain is a sensitive parameter. Quantitative analysis of subendocardium and subepicardium LS provides useful non-invasive information for the layer-specific localization of fibrosis^20^. Furthermore, we performed ROC analysis on myocardial strains in the LGE(+) and LGE(−) groups and concluded that global RS, CS, and LS, subendocardial LS, and subepicardial LS have a predictive value for LGE. That is to say, when these strain parameters are reduced to a threshold in patients with ILVNC, we can predict the possibility of focal myocardial fibrosis in that patient. The variation of these strain parameters suggested that the quality and efficiency of ILVNC myocardial movement in all directions were significantly reduced in patients with LGE.

## Limitations

There are some limitations that need to be considered. Firstly, the sample size is small, and other forms of cardiomyopathy, including dilated cardiomyopathy and valvular cardiomyopathy, can also be manifested as left ventricular enlargement and excessive trabeculation, requiring further comparative analysis. Secondly, LGE could not evaluate diffuse myocardial fibrosis; this requires further study using native T1 and extracellular volume fraction. Thirdly, most patients with ILVNC had a moderate to severe reduction in LVEF. It was not possible to analyze the strain change pattern in patients with preserved LVEF.

## Conclusions

CMR-TT enables the comprehensive evaluation of regional and layer-specific strain. ILVNC is a heterogeneous cardiomyopathy with the most severe apical and subendocardial involvement. Patients with LGE and ILVNC had a higher degree of myocardial strain reduction than those without LGE and ILVNC. CMR-TT can provide more comprehensive information for assessing cardiac function in patients with ILVNC.
